# Piloting a social supervision model in psychiatric placements: A focus group protocol with medical students, residents, and training coordinators (SUPPORT)

**DOI:** 10.1016/j.mex.2026.103847

**Published:** 2026-02-26

**Authors:** Lise-Mari Lauritzen, Edvin Schei, Henrik Myhre Ihler, Madicken Victoria Söderlind Roald, Victoria Akre, Ingrid Amalia Havnes

**Affiliations:** a Division of Mental Health and Addiction, Oslo University Hospital, PO box 4959 Nydalen, Oslo 0424, Norway; b Department of Global Public Health and Primary Care, Faculty of Medicine, University of Bergen, P.O. Box 7804, 5020 Bergen, Norway; c Institute of Clinical Medicine, Faculty of Medicine, University of Oslo, PO box 1171 Blindern, Oslo 0318, Norway

**Keywords:** Medical education, Clinical supervision, Competency-based education, Internship and residency, Psychiatry, Intervention, Qualitative, Focus groups, Collective thematic analysis

## Abstract

Psychiatry faces global recruitment challenges and is often viewed as low prestige among medical students. From their perspective, prior negative experiences including poor supervision and exclusion may reduce engagement during psychiatric placements. At the University of Oslo (UiO), students are typically supervised by residents, yet formal supervision training comes late and lacks practical components despite being required for specialist qualifications. This study will pilot a socially grounded supervision model emphasizing social safety as a driver of supervisory competence and learning outcomes.

Phase 1 will test the feasibility of organizing residents and students into *supervision families* at Oslo University Hospital, deliver a supervision seminar for residents and offer targeted resources to enhance student engagement. Phase 2 will explore experiences with and without supervision families through interviews with students, residents, and training coordinators, assessing perceptions of intervention components and identifying barriers and facilitators. Phase 3 will implement a revised version of the intervention including updated seminars and improved learning resources, including a digital communication skills simulator, and re-examine new student and resident participant experiences to compare perceived impact. Data will be analyzed using a collective thematic approach. Findings will inform refinement and implementation of the model in psychiatric training for UiO students.

• Pilot a socially grounded supervision model during psychiatric placements aiming at strengthening residents’ supervisory competence and enhance student learning.
• Explore experiences and barriers through focus group interviews with students, residents, and training coordinators.
• Use findings to refine and inform implementation of the model for medical students and residents in mental health and other clinical settings.

## Graphical abstract

**Figure:**
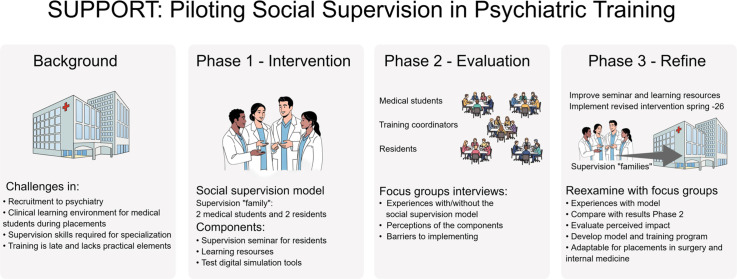

**Specifications table**

**Table:** 

**Subject area**	Medicine and Dentistry
**More specific subject area**	Medical education
**Name of your protocol**	Piloting a Social Supervision Model in Psychiatric Placements: A Focus Group Protocol with Medical Students, Residents, and Training Coordinators (SUPPORT)
**Reagents/tools**	Not Applicable
**Experimental design**	Not Applicable
**Trial registration**	Not Applicable
**Ethics**	The research will be conducted in accordance with the guidelines of the Declaration of Helsinki. The Data Protection Officer for Research at Oslo University Hospital has approved the study (Ref. 25/20251). All the study participants will receive thorough information about the study in advance, both orally and in writing. Written informed consent will be obtained from all participants. Medical student participants will be reimbursed for their time and contribution to the study. Participation is entirely voluntary, and withdrawal from the study will have no negative consequences.
**Value of the Protocol**	• This study addresses a critical gap by exploring a socially grounded supervision model in psychiatric placements, an area with limited prior research. • The study will generate insights into how a socially structured supervision model and learning tools are experienced by medical students and residents, informing refinement to strengthen learning outcomes and supervisory competence. • The study may help identify organizational barriers and enablers for implementing new supervision models within complex hospital systems, informing future strategies for integration and improvement.

## Background

Psychiatry has long faced recruitment challenges worldwide, contributing to a shortage of psychiatrists and a gap between the need for care and available services [1,2]. These challenges may be linked to attitudes towards mental illness, as medical students often hold negative biases toward mental illness before placement [3,4] and frequently describe psychiatry as low-prestige and involving the most challenging patients [5].

Attitudinal challenges also intersect with structural constraints in clinical training. Luhrmann’s ethnographic study [6] depicts clinical placement as unsettling, sometimes marked by exposure and humiliation – though less so in psychiatry than other specialties. Recent research shows that relational omissions, such as physicians failing to greet or include students, can evoke shame, stigma, and feelings of alienation, undermining psychological safety and restricting participation [7,8]. Supervision quality during clinical placements varies considerably across sites, with unclear roles, lack of formal training, and time constraints, contributing to inconsistent support [[9], [10], [11], [12]]. Effective workplace learning depends on acceptance of the learner role, affective support, and high-quality supervision – elements that are often absent in emotionally cold environments marked by shame and weak supervisor contact [8]. Without these structural and relational supports, medical students struggle to achieve intended learning outcomes and to develop a secure professional identity [7].

During psychiatric placements in Norway, medical students are frequently supervised by residents, who are often not trained for the task. Postgraduate medical education in Norway follows a competency-based framework that residents must follow to qualify as specialists [13]. In parallel with medical training, The Common Competency Goals for all medical specialties include objectives across domains such as ethics, communication and patient safety, with clinical supervision and mentoring explicitly emphasized. Although a supervision course is offered, it typically occurs late in specialization and is not directly linked to clinical supervision, raising concerns about its relevance and effectiveness for promoting meaningful learning and supervisory skills during specialization.

Building residents’ supervisory competence and motivation is not only a formal requirement but a strategic opportunity to enhance medical students’ learning and shape their attitudes toward psychiatry. Placements can foster positive attitudes when students encounter supervisors who act as role models, and competent, patient-centered practice can counter stigma and improve perceptions of the field [14]. On the other hand, clinical placements serve a deeper educational purpose: professional identity formation. This process is also driven by identification with role models, typically residents in psychiatry, and can ignite a transformative motivation to think, feel, and act as a psychiatrist, fostering belonging, confidence, and commitment [15].

Based on experience-based learning theory, professional identity formation and participation requires three forms of clinician support: affective, pedagogical, and organizational. These supports foster psychological safety and make guided reflection with a mentor possible, building three core capabilities – intellectual, practical, and affective, that collectively constitute professional identity [16]. However, research on how supervision should be structured to optimize learning and how residents can best develop pedagogical skills remains limited [17], revealing a critical gap in the organization and support of psychiatric placements.

## Description of protocol

### Overall objective

This study and intervention aim to pilot, refine, and evaluate a socially grounded supervision model designed to enhance medical students’ clinical engagement and residents’ supervisory competence during psychiatric placements. The model is based on small *supervision families* where e.g. two residents and two medical students work closely together throughout the psychiatric placement. The intervention also includes a preparatory supervision seminar for residents and digital learning resources for students. The placement will be evaluated both with and without the supervision families using qualitative methods, through focus group interviews with students, residents, and training coordinators. Based on these findings, the supervision model and learning tools will be refined, tested and re-evaluated.

The study will be conducted in three phases, and to ensure coherence across these phases, the analysis is guided by two overarching research questions. These questions establish a unifying analytical framework for Phases 1–3 and clarify how feasibility, perceived impact, learning mechanisms, and supervisory competence will be examined throughout the study:

RQ1: How does a socially grounded supervision model shape students’ and residents’ experiences of social safety, engagement, professional identity formation, and the development of supervisory competence during psychiatric placements?

RQ2: What mechanisms and contextual factors enable or limit the implementation, feasibility, and perceived impact of the supervision families and associated learning resources?

## Specific objectives

### Phase 1 – Pilot a pragmatic intervention

Phase 1 primarily addresses feasibility and early implementation conditions, generating initial indicators relevant to social safety, engagement, and identity development (RQ1) while identifying contextual enablers and constraints (RQ2). The intervention involves piloting a socially grounded supervision model at Oslo University Hospital – testing the feasibility of organizing residents and students in supervision families, and deliver a supervision seminar for residents, a welcome session for students, and produce and distribute a learning video on activation strategies to promote clinical engagement for students. The welcome session will include a brief orientation that emphasizes students’ active role in building supervisory relationships and in offering to participate in clinical tasks where appropriate and safe; this short awareness‑raising element is included because student stance is pivotal for intervention success. The activation strategies include, but are not limited to, engagement with a recently developed online learning resource platform (*psykosekompetanse.no*), which revolves around case-based learning and direct verbal interaction with an AI-powered conversation simulator to practice clinical skills. To address experienced gaps in practical experience during Phase 1, the AI-enabled clinical interaction simulator will be refined to better prepare students for complex clinical encounters, focusing on cases involving severe mental illness such as psychosis and bipolar disorder.

### Phase 2 – Focus group interviews: Explore experiences and barriers

Phase 2 involves an in‑depth exploration of experiences with and without supervision families through focus groups with students, residents, and training coordinators. The phase examines perceptions of the intervention components, gathers suggestions for improvement, and identifies barriers and facilitators to implementing the supervision model. In doing so, Phase 2 elucidates mechanisms that shape learning, supervisory competence, and perceived impact (RQ1), and details barriers and facilitators at individual, relational, and organizational levels (RQ2).

### Phase 3 – Refine and compare

Phase 3 entails implementing a revised version of the intervention in spring 2026, including an updated seminar for residents, welcome meeting with students, and improved learning resources including access to the AI-enabled clinical interaction simulator. Supervision families will be organized, and participant experiences with the supervision model will be re-examined to compare perceived impact. In doing so, Phase 3 evaluates perceived impact and the plausibility of proposed mechanisms (RQ1), alongside practical considerations for broader implementation (RQ2).

Following these phases, the study will inform the development of a supervision model and training program for use in mental health and addiction medicine across hospitals in the South-Eastern Norway Regional Health Authority that host medical students. The model will also be applicable to placements in internal medicine and surgery.

### Theoretical framework

The study is grounded in experience-based learning theory, where students’ participation in communities of practice is essential. Students must be granted legitimate peripheral participation – accepted as potential members in a peripheral role requiring adaptation and support. As students gain knowledge and autonomy, their position within the community becomes increasingly central. Participation turns students from passive observers into active members of clinical teams. Through supported involvement in real patient care, they develop capability and professional identity, linking theory to practice. Professional identity formation, participation, and guided reflection with a mentor depend on three forms of clinician support: affective, pedagogical, and organizational. These supports foster professional identity through three core capabilities – intellectual, practical, and affective [16,18].

Complementing this perspective, sociocultural learning theory posits that clinical competence develops through collaboration with a more experienced professional – a more competent other. Clinical placements should therefore provide tasks situated within the student’s zone of proximal development: activities that are too complex to complete independently yet achievable through guided interaction with a knowledgeable supervisor [19]. This also aligns with the traditional apprenticeship approach, where the student learns while participating in the workplace [20].

Kvernenes et al. [21] further underscore the relational dimension of workplace learning. Basic social interaction can carry significant pedagogical weight, influencing students’ sense of belonging and professional identity formation. The concept of *phatic* interaction rituals highlights how greetings and recognition function as essential signals of inclusion and respect. Without these signals, students may become passive, avoid engagement, and question their suitability for a medical career [7]. Ribeiro and de Carvalho Filho [22] argue that student-centered education and patient-centered care are deeply interconnected: the way we educate medical students shapes their future clinical practice. When education is hierarchical, standardized, and fragmented, it risks reproducing the very problems that patient-centered care seeks to overcome.

Additionally, it is important to consider practical frameworks that address the structural and organizational conditions for effective clinical education. In this regard, the intervention draws on key success factors identified by Caspersen and Kårstein [12], which – although not forming the theoretical basis of the study – offer valuable insights into the design and implementation of clinical placement. These factors include relevant and practice-oriented supervisor training, explicit delegation of responsibilities, clear communication of expectations to students, systems for quality assurance and competence development.

This theoretical and practical foundation aligns with the principles of competency-based medical education, which require learning environments that allow students to demonstrate competence in authentic clinical settings [13]. Within this framework, supervision plays a critical role – not only in supporting academic development, but also in fostering trust and evaluating readiness for greater autonomy.

### Context

The study is situated at Division of Mental health and Addiction, Oslo University Hospital (OUH) and involves collaboration with University of Oslo (UiO) – one of four universities within the Norwegian undergraduate medical education system. OUH provides inpatient, day-care, and outpatient services across multiple sites in psychiatry, child and adolescent psychiatry and addiction medicine. OUH has 63 designated psychiatry residency positions, and it is mandatory to supervise medical students at least once during residency. To qualify as a supervisor, residents are expected to have a minimum of two years of clinical experience in psychiatry. The study is anchored in the hospital’s strategic plan to recruit and retain healthcare professionals.

The study will include fourth-year medical students enrolled under the previous admission structure, approximately 110 students twice a year. From 2026 the annual intake will increase to about 275 students, distributed across two semesters. The 8th semester covers psychiatry, addiction medicine, forensic medicine, and pharmacology through lectures, seminars, and weekly small-group sessions where students meet patients with different diagnoses under the supervision of psychiatrists holding combined academic and clinical positions at UiO and OUH. This module includes the students’ first extended clinical placement in the program; a three-week rotation in psychiatry at a single clinical site per student, including placements in child and adolescent psychiatry and addiction medicine.

Clinical placements are allocated through a ranked selection system, allowing students to choose available sites in turn based on their assigned number. Up to 50 students can select their psychiatry placement at OUH, while the remaining students choose other hospitals within the South-Eastern Norway Regional Health Authority, which serves 3.1 million inhabitants that accounts for 57 % of Norway’s population.

### Pragmatic intervention

The intervention is designed as a pragmatic approach, building on principles from the international PROFMED project, which promotes professional identity formation through structured preparation for clinical placements and faculty development for supervisors [21]. In our study, these principles are adapted to psychiatric placements, including efforts to organize participants into supervision families. Due to the complexity of OUH, with multiple inpatient wards and outpatient clinics distributed across several geographical locations, practical constraint underpins the term *pragmatic intervention*. Beyond grouping students and residents together as families, the intervention targets both groups separately through tailored activities.

### *The size of the supervision family*

Although the ideal configuration is two students and two residents, practical, geographical, and competence‑related considerations necessitate a pragmatic approach. Consequently, a supervision family may in some cases consist of one to three students and two to three supervisors and may include a specialist when residents are at an early stage of their specialist training. Within this flexible framework, a small group remains preferable to support social safety, while also enabling peer learning and shared supervisory workload.

### Digital learning tools

For students, the intervention includes user involvement through co-development of a learning video highlighting active engagement during clinical practice. The video is informed by previous placement evaluations and by insights from 16 student interviews conducted by a fellow medical student and will be distributed prior to the placement. This will be complemented by a digital welcome session providing practical information and an introduction to learning opportunities, including the online interactive platform *psykosekompetanse.no*. This is designed to support case-based learning across a broad continuum of severe mental disorders. The platform encompasses several key domains, including diagnostic assessment, therapeutic interventions, family and caregiver involvement, and professional communication skills. Educational content is delivered through multiple modalities – written materials, video lectures, and podcasts – and is organized around nine clinically representative personas. This design facilitates reflective practice and discussion of clinically relevant scenarios.

In addition to passive learning, the platform offers interactive components aimed at enhancing clinical competencies. The medical students will be invited to engage in direct verbal dialogues with the personas through an AI-powered conversation simulator, to practice communication skills, symptom recognition, and admission interview techniques. These simulations are intended to complement, rather than replace, direct patient interaction during clinical placements, thereby providing opportunities for active learning between patient encounters. The AI-driven conversations are scored and transcribed and can be used directly in supervision sessions with residents.

### Supervision seminar

For the psychiatry residents, the intervention includes a full-day supervision seminar organized by the research team immediately before the placement period. The seminar introduces the PROFMED supervision model and is designed to foster social safety, reflection, and active engagement. Key components include:

1. Activities to get to know each other and build a supportive learning environment – paired interviews to reflect on supervision during placement, emphasizing personal experiences, possible learning outcomes, and valuable approaches to creating trust in student-supervisor relationships.
2. An overview of theory on the development of the students’ professional identity and mastery of the physician role through clinical placement.
3. Role-play illustrating different student types and approaches to supervision in emotionally challenging situations.
4. Practical suggestions for stimulating learning activities and student responsibilities tailored to various clinical settings, informed by experiences from resident supervisors and interviews with previous students after the placement period (see Table 1).

**Table 1:** Learning activities.

Category	Examples of Learning Activities
Patient Contact and Independent Work	Observe and conduct admission interviews; participate in suicide risk assessments.
Writing and Documentation	Write admission assessments and discharge summaries; document psychiatric status; receive feedback on written notes.
Feedback and Reflection	Receive specific feedback on patient consultations; discuss patients’ psychiatric history, diagnosis and treatment plans with resident/supervisor.
Interdisciplinary Collaboration	Participate in interdisciplinary meetings; collaborate with and learn from other professional groups through observing their encounters with patients; attend meetings with relatives and external agencies.
Communication and Presentation	Present assessments in team meetings; explain legal frameworks and treatment plans; develop and present care plans.

## Qualitative evaluation

The intervention will be evaluated using qualitative methods – focus group interviews with medical students, resident supervisors, and local coordinators responsible for organizing placements at OUH. This exploratory design allows in-depth examination of experiences, attitudes, and perceived barriers to implementation [23]. Medical students will be invited to discuss their experiences from the placement, including the welcome session, learning video, and supervision arrangements. Residents will be asked about their participation in the supervision seminar and their role in supervising students during current and previous placements. To identify organizational challenges, training coordinators and clinical leaders will also participate in focus group interviews. Separate, homogeneous focus groups will be employed to minimize power asymmetries between students, residents, and coordinators, and to ensure privacy and confidentiality [24]. Two to three researchers from the research group will attend the interviews, with one researcher acting as the moderator and ensuring active participation while the others contribute follow-up questions.

To ensure that the concept of social safety is grounded empirically, the analysis will attend to several observable and reported indicators. Social safety will be identified through: 1) interactional markers, such as greeting practices, inclusion in conversations, opportunities to participate in clinical tasks, and supervisors’ openness to questions; 2) reported emotional experiences, including feelings of being welcomed, respected, anxious or exposed; and 3) participation patterns, such as active involvement, withdrawal, silence, or avoidance. These indicators will guide coding and interpretation across all focus group data and enable a nuanced examination of how social safety is enacted in practice.

## Participants and recruitment

### Phase 2: Explore experiences and barriers – inclusion criteria focus group interviews

Recruitment will aim for broad perspectives on the placement experience, seeking representation of medical students, psychiatry residents, and training coordinators – the latter may also serve as a senior consultant, leader, or supervisor. Recruitment of interviewees will not depend on attendance at the supervision seminar, the welcome meeting, or membership in a supervision family. We plan to conduct two focus groups with medical students, two focus groups with psychiatry residents, and one focus group with senior consultants who serve as site coordinators, each consisting of approximately 4–6 participants to promote active engagement and productive discussion. Groups will be interviewed once, with a maximum duration of 1.5 h.

### Phase 3: Refine and compare – inclusion criteria for focus groups

Medical students and psychiatry residents who have been organized in supervision families during the placement first semester 2026 will be included: two focus groups with medical students, and two focus groups with psychiatry residents, each consisting of 4–5 participants. Site coordinators will not be included in this phase.

Recruitment will involve multiple strategies tailored to each target group. Medical students will receive written invitations via email and SMS, and information will also be shared in the cohort’s Facebook chat by student assistants, who will actively disseminate details among peers. In addition, members from the research team plan to attend the end of a scheduled lecture at UiO to present the project. Psychiatry residents will be informed orally during the supervision seminar and mandatory weekly teaching sessions as part of their structured specialist training program, in addition to receiving written invitations via email and SMS with a link to Microsoft Forms for selecting available time slots. If recruitment for focus groups is unsuccessful, individual interviews will be offered. Coordinators will be invited via e-mail, SMS and phone calls, with digital interviews offered to lower the threshold for participation.

### Informational power

We will assess data adequacy using the concept of informational power: When the study aim is focused, the sample is specific, the interview dialogue is rich, and the analytical approach is coherent, fewer participants are needed. When these conditions are weaker, more participants are required [25]. We will judge sample sufficiency iteratively within each stakeholder group by monitoring the fit between participants and the aim, relevance of emerging material, and analytic progress. Decisions to stop inclusion will be documented.

### Resident variability and bias reduction

To contextualize findings and mitigate potential bias arising from variability among residents, we will collect a brief set of background characteristics from participating residents at the start of the interviews (years in psychiatry, prior supervision experience and teaching motivation). During the collective thematic analysis, such variability will be treated as analytically relevant and explicitly explored in relation to experiences of facilitation, supervisory practices, and perceived learning outcomes. Variation will further be addressed through data and researcher triangulation – separate focus groups for students, residents, and coordinators, and an analysis team comprising students, a graduate, a resident, and experienced researchers, thereby reducing the risk that any single resident profile disproportionately shapes interpretations [26].

### Data collection

The interviews will be conducted sequentially: first with medical students, then psychiatry residents, and finally with leaders. The interview guides are structured as topic guides [24], and will be revised iteratively based on insights from previous interviews, in accordance with data source triangulation – a collection of information from different types of people to gain multiple perspectives on a phenomenon [26], and to enable comparative analysis across focus groups [24].

### Topic guide

The topic guides are organized around overarching themes informed by principles of affective, pedagogical, and organizational support [16]. In addition to these themes, participants are encouraged to engage in open discussion, with the moderator posing follow-up questions as needed. The guides are developed by researchers within the research group including student research assistants, an approach inspired by researcher triangulation, aimed at capturing breadth through diverse perspectives and interpretations, and at reducing interpretative bias and increasing validity [26]. Members of the research group have backgrounds as psychiatrists, senior consultants, union representative for residents, and lecturer at OUH, University of Bergen and UiO, alongside other members specializing in pedagogy and teacher education. Edvin Schei developed the method used in PROFMED [21], which has informed the socially grounded supervision model applied in this study.

The themes included in the topic guide are summarized in Table 2.

**Table 2:** Themes.

Participants group	Main themes
Medical students	- Reception at clinical site and impact of welcome meeting - Suggestions for improving start of placement - Reflections on what worked well or less well - Learning activities, perceived outcomes, engagement - Attitudes toward psychiatry before, during, and after placement - Reflections on how a supervision family might influence placement
Resident	- Reflections on supervision seminar - Experiences from placement period - Supervision techniques and learning activities - Motivating or rewarding aspects of supervision - Reflections on what supervision families could provide
Leaders/senior consultants	- Awareness of project in hospital’s strategic plan - Collaboration to optimize placement - Perspectives on who can supervise students - Needs for planning and information flow - Characteristics of a good learning environment - Experiences with recruitment following placement

### Collective thematic analysis

The data will be analyzed using collective thematic analysis, as described by Eggebø [27]. This approach involves convening key stakeholders for joint analysis sessions to foster a creative, rigorous, and transparent interpretation of the material, also an approach in accordance with researcher triangulation [26]. In this study, the analysis group will include researchers with various backgrounds, and one resident physician, one graduate, and three medical students. As the study aims to improve psychiatric placements, it is essential that interpretations reflect the perspectives and needs of both students and residents. Researchers will contribute by transcribing the interviews before the analysis and prepare anonymized summaries. Further on they will moderate the analysis-workshops and safeguard research quality throughout the process. They will also provide pedagogical, clinical and theoretical insights and uphold ethical and methodological rigor.

The students, the graduate and the resident will provide experiential viewpoints from their respective roles in the analytical phase. The students and the graduate will focus on empirical reading without applying theoretical frameworks, challenge assumptions, and highlight issues relevant to student experiences. They will bring a fresh, learning-oriented perspective and question established views. The resident will contribute clinically oriented insights and emphasize themes relevant to practice and supervision, and bridge theory and clinical practice to ensure practical relevance. Incorporating multiple perspectives enhances reflexivity in qualitative research [24]. Allowing others to examine the material and offer alternative interpretations helps researchers critically reflect on their own preconceptions, assumptions, and theoretical positions.

Consistent with this stance, the collective thematic analysis will be primarily data‑driven during coding and initial theme development, without predefined theoretical categories. The theoretical framework will then be used in later interpretive stages to contextualize and deepen the empirically derived themes. Thus, we combine inductive theme generation with theory‑informed interpretation, keeping these steps analytically distinct [27].

### Reflexivity

We recognize that researchers always enter the field with pre‑existing lenses, formed by prior experiences, assumptions, professional roles, and theoretical commitments. Such preunderstandings may productively motivate inquiry, but they can also narrow attention and make expected patterns easier to see than discrepant evidence [28]. To address this, we will maintain a reflexive stance throughout the study. The research team acknowledges that its diverse professional positions (students, a resident, senior clinicians) may influence both data collection and interpretation. Reflexivity will therefore be integrated into the collective thematic analysis through ongoing discussion of assumptions and potential biases, and through ongoing reflexive discussions in which team members explore and compare their readings of the material. This process aims to make the analysts’ perspectives visible, prevent any single viewpoint from dominating, and strengthen the credibility of the resulting themes.

## Analytical procedure

The collective analysis will follow four steps outlined by Eggebø [27]:

1. Joint Review of Data: The group will review anonymized summaries of interview transcripts to gain an overview of the material. Full transcripts will be available for reference. Summaries will be prepared by the moderator prior to the session.
2. Theme Identification: All participants will independently note potential themes, ideas, and analytical questions.
3. Theme Grouping: Through discussion, themes will be organized into main categories and subthemes.
4. Outline and Work Plan: The group will draft article outlines, assign writing responsibilities, and plan subsequent analytical and writing tasks.

The digital communication skills simulator is an intervention designed to enable learners to practice patient encounters and receive structured digital feedback. It is distinct from the socially grounded supervision model, as it focuses specifically on individual skills training that can be undertaken independently. Consequently, data generated through the simulator can be coded and analyzed separately. The resulting analyses will be used to explore two separate domains of interest: 1) perceived learning outcomes, learners’ preparedness for patient encounters, and general satisfaction with the simulator as a learning activity in psychiatric placements, and 2) to inform further refinement and development of the communication skills simulator.

All participants may engage in all four steps of the analysis depending on availability. Participants’ contributions will be preserved and contextualized within the full dataset to ensure validity and comprehensive interpretation [29]. The results from Phase 2 will be used to refine the intervention and to improve and develop learning resources tailored to situated learning, as well as affective, pedagogical, and organizational support. The results from Phase 3 will be utilized in scientific publications and will contribute to quality improvement in psychiatric practice, as well as inform a supervision model and resource package to be distributed to hospitals within the South-Eastern Norway Regional Health Authority that host medical students.

### Ethical considerations

The study will comply with the Declaration of Helsinki and the ethical guidelines of the Norwegian National Research Ethics Committees. The project is not classified as health research under the Health Research Act and therefore does not require approval from the Regional Committees for Medical and Health Research Ethics (REK). Approval has been obtained from the Data Protection Officer at Oslo University Hospital (25/20251) as participants are employees or medical students undertaking clinical placement.

Participation is voluntary and based on informed consent. All participants will receive oral and written information and sign a consent form prior to inclusion. They may withdraw at any time without providing a reason and without consequences; in such cases, personal data will be deleted unless already included in analysis or publications. Invitations will be neutral and free from undue pressure. Medical students will receive a gift card of 50 EURO to compensate for time spent.

Each interview will be audio-recorded, and the audio recordings will be made using hospital-approved devices and transferred immediately to secure storage; recordings will be deleted after transcription. Transcriptions will be anonymized, and personal identifiers removed. Confidentiality will be ensured; no individuals or clinical sites will be identifiable. Data will be securely stored on access-restricted servers in accordance with privacy regulations. Quotes and descriptions will be anonymized to prevent direct or indirect identification. A separate encrypted key linking participant ID to personal data will be stored securely and deleted no later than five years after project completion. Participants have the right to access and correct their data.

### User involvement

The study adopts a strong user-oriented approach. In spring 2025, one medical student contributed to exploring student experiences and preparing learning activities and content for the instructional video as part of the study planning. Mentimeter evaluations of residents’ supervision experiences with medical students informed the design of the intervention. Both residents and medical students are actively engaged in the project by participating in the collective thematic analysis of the data and contributing to the development of the interview topic guide to ensure relevance and alignment with student and resident perspectives. Furthermore, the guides draw on evaluations and feedback from residents and medical students, including interviews with 16 students following the spring 2025 placement period.

The project team comprises three medical students and one graduate, one resident, and one senior consultant, board-certified at Oslo University Hospital, all employed as hourly paid research assistants.

## Protocol validation

Not applicable

## Limitations

This qualitative study is based on focus group interviews, which may introduce contextual effects, as group dynamics and social relationships can influence what participants choose to share or withhold [24]. Sensitive experiences, such as exclusion, evoking shame during clinical placement [8], which are central to the study’s theoretical framing, may not be disclosed in a group setting. To mitigate this limitation, the study uses homogeneous focus groups to reduce power asymmetries [24], and the moderator will employ an open, supportive facilitation style to encourage reflection on challenging experiences while ensuring psychological safety. Using the same moderator for all sessions can also reduce interviewer-related variation. The study is designed as a pragmatic pilot, and adaptations during implementation (e.g. flexible scheduling, additional seminars) may introduce variation in how the model was delivered, affecting consistency and comparability.

## Standardized qualitative research reporting

We aligned the protocol with SRQR principles [30] and consulted relevant COREQ items where applicable [31]. The choice of reporting framework for the results paper will be made when empirical findings are available.

## CRediT author statement

**Lise-Mari Lauritzen**: Conceptualization, Methodology, Investigation, Data Curation, Writing – original draft, Formal analysis, Writing – review & editing, Visualization, Project administration.

**Edvin Schei**: Conceptualization, Methodology, Investigation, Formal analysis, Writing – review & editing, Supervision.

**Henrik Myhre Ihler**: Methodology, Resources, Investigation, Formal analysis, Writing – review & editing.

**Madicken Victoria Söderlind Roald**: Methodology, Writing – review & editing.

**Victoria Akre**: Conceptualization, Methodology, Writing – review & editing.

**Ingrid Amalia Havnes**: Conceptualization, Methodology, Investigation, Resources, Data Curation, Formal analysis, Writing – original draft, Writing – review & editing, Visualization, Supervision, Project administration, Funding acquisition.

## Supplementary material *and/or* additional information [OPTIONAL]

Not applicable

## Declaration of competing interest

The authors declare that they have no known competing financial interests or personal relationships that could have appeared to influence the work reported in this paper.

## Data Availability

Qualitative data from focus groups cannot be shared due to confidentiality. Anonymized topic guides and the analytic framework will be available upon reasonable request from the corresponding author.
